# Beyond boundaries: Redefining the donor frontier in pediatric lung transplantation

**DOI:** 10.1016/j.jhlto.2025.100355

**Published:** 2025-07-25

**Authors:** Darren Turner, David L. Morales, Don Hayes

**Affiliations:** The Heart Institute, Cincinnati Children’s Hospital Medical Center, University of Cincinnati College of Medicine, Cincinnati, Ohio

**Keywords:** pediatric, lung transplant, organ donor, deceased after cardiac death, EVLP, ex vivo lung perfusion, living donor lobar lung transplant

## Abstract

**Background:**

Lung transplantation remains the optimal treatment for children with end-stage lung disease, yet donor organ shortage represents the greatest obstacle to transplantation. In 2023, only 31 pediatric lung transplants were performed in the United States, with 9% of recovered lungs ultimately not transplanted. Pediatric waitlist mortality has increased, particularly for patients under one year of age, necessitating innovative strategies to expand the donor pool.

**Methods:**

This review examines emerging strategies to combat organ shortage in pediatric lung transplantation, including extended criteria donors, deceased cardiac death (DCD) organ donation, ex-vivo lung perfusion (EVLP), graft size reduction techniques, living donor lobar transplantation, and utilization of hepatitis C and HIV-positive donor organs. We analyzed current literature and clinical outcomes data to assess the feasibility and safety of these approaches in pediatric populations.

**Results:**

Extended criteria donors now account for 80% of lung transplants without compromising short- and mid-term pediatric outcomes. DCD lung transplantation demonstrates comparable survival rates to brain-dead donors, with only 14 DCD organs used in pediatric programs between 2004-2022. EVLP shows promise in preserving organ viability and reducing primary graft dysfunction. Hepatitis C-positive donors demonstrate excellent outcomes with direct-acting antiviral therapy in adult patients, but scant literature is available in the pediatric population. Reduced-size grafts and living donor procedures offer solutions for size-mismatched recipients.

**Conclusions:**

Multiple innovative strategies show potential for expanding the pediatric lung donor pool. While adult data demonstrates safety and efficacy, pediatric-specific research remains limited. Continued scientific inquiry, active donor management protocols, and interdisciplinary cooperation are essential to safely implement these approaches and improve access to life-saving transplantation for children.

## Background

Lung transplantation remains the best treatment option for children with end-stage lung disease. In 2023, 31 pediatric lung transplants were performed in the United States, and globally, more than 2,500 lung transplants have been performed worldwide.^1^, ^2^ However, one of the greatest obstacles to transplantation is a shortage of adequate donors. In 2023, 9% of lungs recovered for transplant were ultimately not transplanted.^1^ Despite composite allocation scoring and other similar policies, a paucity of donors remains, particularly in the pediatric population. Moreover, pediatric waitlist mortality has unfortunately increased in 2023, most notably for those under the age of 1 year.^1^ Several strategies have emerged to combat waitlist mortality, including the use of extended criteria organs, the use of deceased cardiac donors, ex vivo lung perfusion (EVLP) techniques, graft size reduction, and living donors. In this review, we will discuss these strategies that could allow the expansion of the pediatric donor pool for lung transplantation.

## Extended criteria donors

One of the most significant strategies to increase the donor pool was the introduction of extended criteria organ donors. Due to the restrictive nature of standard lung donor criteria and the need for organ donors, there has been increased interest in organs that would fall outside of the criteria, called extended criteria donors. Standard criteria include donor age <55, a normal chest X-ray, paO_2_ >300 mm Hg, <20 pack-years smoking history, absence of chest trauma, malignancy, or microorganisms, and no purulent secretions or signs of aspiration.^3^ The use of these organs significantly increased during the 1990s, and currently, 80% of lung transplants are performed with extended criteria donors.^4^ Furthermore, their use has not compromised short- and mid-term outcomes in pediatric patients.^5^ However, these extended criteria donors should be used cautiously, as use in high-risk patients (lung allocation score ≥70) has been associated with reduced 1-year survival.^6^

## Deceased cardiocirculatory death organ donation

Organ donation from deceased after cardiocirculatory death (DCD) has been increasingly used in solid organ transplant to combat the organ shortage in adults. With these donors, procurement and timing of transplantation are key. After withdrawal of life support, the donor is allotted 60 minutes for cardiac arrest to occur. Should arrest occur, there is a mandatory “hands-off” phase of 5 minutes to ensure no autoresuscitation occurs. Afterward, death is declared, and the patient is taken to the operating room. Because of this relatively quick turnaround, the transplant center and organ procurement organization must be in constant communication to minimize ischemic time.

In the case of lung transplant, deceased after brain death (DBD) donors can be deemed unsuitable for transplant due to pneumonia, pulmonary edema, trauma, and other effects of brain death on the lungs.^7^, ^8^, ^9^ Moreover, lung tissue does not solely rely on perfusion for cellular respiration, as most of the cells in the lung are exposed to air, highlighting the potentially less deleterious effect of cardiocirculatory death on lung tissue quality.^10^ Still, the number of DBDs used is only increasing because of the opioid crisis.^11^

Indeed, the first successful attempt at lung transplant utilized a DCD donor.^12^ Due to the popularity of DBD, the use of DCD organs dramatically declined. In 1991, Egan et al conducted a series of experiments to reintroduce the use of DCD donors.^10^ Since then, its use has been slowly increasing. As of 2020, a total of 728 adult DCD lung transplants have been performed in the United States, with 528 of transplants occurring between 2015 and 2020, indicating only recently increased popularity.^13^ Unfortunately, the practice of DCD organ procurements has been slow to gain widespread acceptance, likely due to logistical challenges and some ethical concerns.

Outcomes in DCD lung transplantation are encouraging. A large meta-analysis conducted by Krutsinger et al demonstrated that there was no difference in survival after lung transplant from DCD donors vs DBD donors.^14^ Studies of the International Society for Heart and Lung Transplantation Registry also showed similar results with comparable 5-year survival rates.^15^ Unsurprisingly, there are significantly fewer pediatric lung transplants as compared to adults. And as expected, there is even less utilization of DCD lungs in candidates among all pediatric programs in the United States, with only 14 DCD organs having been used in pediatric heart or lung transplantation between 2004 and 2022.^16^ Due to recent literature reporting positive outcomes for children after DCD lung transplants, our program has become receptive to DCD lungs.^17^

Strategies to increase DCD donor use have included protocols to standardize procurement, procurement questionnaires, and team education, but much work is to be done to increase DCD lung donation. This may present the most significant opportunity to increase access to lung transplantation, as some estimates conclude that DCD organ use would provide an additional 22,000 annual donation opportunities worldwide.^18^

## Ex vivo lung perfusion

Supplementing DCD procurements is the advent of EVLP, which has helped combat damage of warm ischemia and sustain organ viability. In adult lung transplant (LTx), it has been reported that 12% of DCDs were performed using EVLP.^15^ A large study performed by Machuca et al^19^ reported that 9% of their 673 DCD lung transplants were performed with EVLP, and compared to no EVLP, these recipients had shorter hospital stays and trended toward less time mechanically ventilated. A study by Sanchez et al reports similar outcomes of DCD organs with and without EVLP while also highlighting the utility of EVLP as an adjunct to screen high-risk lungs for viability.^20^ We were only able to identify 7 instances in United Network for Organ Sharing as having used machine perfusion in pediatric lung organ donors as of 2024.

## Graft size reduction and living donor lobar transplantation

A unique challenge in the pediatric population is the frequency of donor-recipient size mismatch.^21^ Size mismatch can result in retention of secretions, atelectasis, infections, persistent pneumothorax, and bronchiolitis obliterans.^22^, ^23^, ^24^ To combat this challenge, techniques have been developed to utilize adult grafts and perform anatomic reductions to make them amenable for pediatric transplant. The most common techniques for reduction involve peripheral nonanatomical resection, lobar resections, and split lung transplants. Small studies have demonstrated that reduced-sized grafts have been used with success in adolescents, young, and even small children.^22^, ^25^, ^26^

Living donors are also an option in centers with the appropriate experience in this procedure. Traditionally, living donor lobar lung transplant (LDLLT) uses a healthy lobe from 2 donors and places them into 1 recipient. Other techniques have also been described, including inverted transplantation and split donor techniques.^27^, ^28^ A major drawback to traditional LDLLT is the labor intensity, requiring 3 operating rooms with 3 different teams to coordinate a single transplant, as well as cost concerns. Furthermore, LDLLT is infrequently performed in the United States, with the majority of these being performed in Japan, likely due to the challenges for DBD donors in that country.^29^

## Hepatitis C and HIV-positive organ donors

Nearly 39.9 million individuals worldwide are infected with human immunodeficiency virus (HIV), with 1.4 million being between the ages of 0 and 14.^30^ Furthermore, over 70 million individuals are chronically infected with the hepatitis C virus (HCV).^31^ Due to major advancements in antiretroviral therapies, patients with HIV have started to live with almost normal life expectancies with suppressed chronic infection, and patients with HCV are experiencing relatively high cure rates.^32^, ^33^ As such, these patients are developing similar end-stage organ disease as those without chronic HCV or HIV. With concomitant changes in transplantation or organ allocation policy and the Hope Act of 2015, many patients infected with HIV or HCV in the United States have become not only recipients of organs but also potential organ donors. And the need for organs has increased interest in these deemed “high-risk” organs. Radzi et al showed that from 2008-2015, over 2,200 high-risk HCV donors were not utilized for pediatric thoracic transplantation.^34^

Studies have consistently demonstrated the safety and feasibility of HCV-positive lung donors to HCV-negative recipients. In 2015, a retrospective study by Bansal et al demonstrated similar survival outcomes of patients who underwent lung transplants from high-risk donors as compared to those who underwent a lung transplant from non–high-risk donors.^35^ In 2019, a large prospective trial by Woolley et al demonstrated the feasibility of heart and lung transplants from HCV-infected donors to uninfected recipients, demonstrating the efficacy of direct-acting antiviral agents at the time of transplant. They noted that although viral load was detected directly after transplant, with adequate treatment, patients became undetectable at 2 weeks post-transplant. Although there were higher rates of rejection in this population, 6-month graft survival was 100%, with all patients having excellent graft function.^36^ There is a paucity of large studies in pediatric lung transplantation, but there is a report of a successful HCV-positive donor lung transplant in this population.^37^ A large study of pediatric kidney transplant showed promising results, but they noted that HCV-positive donors are scarcely used in the pediatric population.^38^ Similarly, the use of HIV-positive donor organs has yielded excellent results, comparable to outcomes of HIV-negative donor organs.^39^ There are even some case reports detailing HIV-positive donor organs used in pediatrics.^40^, ^41^ Still, there is more work to be done to establish the risk profile, antiviral, and immunosuppressive protocol in pediatric patients before we can safely tap into this resource. Moreover, the stigma associated with HIV and HCV organs will only diminish with time.

## Potential clinical impact of donor lung approaches in children

With any of the above donors, certain preoperative and perioperative precautions should be taken to improve transplant outcomes. These procedures are coined as active donor management and encompass several key strategies. Protective ventilatory strategies include limiting tidal volumes to lower driving pressures, optimizing positive end-expiratory pressure, and use of recruitment maneuvers, which can reduce ventilator-associated lung injury.^42^ Perioperative bronchoscopic evaluation of the airways can be performed to not only clear secretions but also look for signs of aspiration.^43^ What is more, one may collect lavage samples at this time. One may also consider the use of corticosteroids to improve oxygenation at organ recovery.^44^ Finally, a conservative fluid management approach should also be used.^45^

Given the novelty of these donor approaches, there are limited data to determine their short- and long-term outcomes in the pediatric population. It is well known in adults that DCD lungs are associated with a higher incidence of grade 3 primary graft dysfunction (PGD).^46^ Prevention of PGD is important because it correlates with inferior outcomes due to heightened mortality, acute and chronic rejection.^47^, ^48^ PGD is characterized by hypoxemia and the presence of lung opacities not explained by other causes, typically occurring within the first 72 hours of transplant.^49^ The physician should be cognizant of strategies to optimize outcomes by decreasing rates of PGD or reducing its impact on the newly transplanted organ. Moreover, LDLLTs are inherently a higher risk population due to increased likelihood of instability and presumably would experience more PGD.^50^ Key strategies for these populations include early detection of PGD and rapid treatment if severe, with early initiation of extracorporeal membrane oxygenation (ECMO) and the use of EVLP.

Early initiation of ECMO in patients with PGD or prior to onset is associated with better survival. A large single-center study revealed a 90-day, 1-year, and 5-year survival for lung transplant recipients who required ECMO for PGD was 67.3%, 50%, and 31.5%, respectively. They also concluded that early initiation of ECMO (<48 hours) was associated with lower mortality.^51^ Barring any hemodynamic or right ventricular dysfunction, veno-venous ECMO is the preferred type of ECMO as a lung allograft protection strategy.^52^

Another strategy is the use of EVLP for the transplantation of the donor organs. Normothermic EVLP has been shown to preserve the metabolic function and homeostasis of the lung to reduce insult from ischemia-reperfusion injury and reduce warm ischemic time, which both contribute to PGD.^53^, ^54^ There are 3 standardized protocols for EVLP: Lung, Toronto, and Organ Care System (TransMedics, Andover, Mass). These three systems differ in perfusate used and pump type, but they can all be used to assess, rehabilitate, and re-evaluate the lung before transplantation.^55^ At the author's institution, we have conducted 3 lung transplants with excellent results using the TransMedic OCS, and with that, we use blood as the perfusate.

Early data suggested that with a decrease in PGD with the use of EVLP, survival and functional outcomes were similar to transplantation without EVLP.^56^, ^57^ This phenomenon was further explored with the Normothermic Ex Vivo Lung Perfusion as an Assessment of Extended/Marginal Donor Lungs trial, which was the first Food and Drug Administration-mandated multicenter, prospective clinical trial. This study showed that PGD results were equivalent between patients who underwent EVLP-treated LTx and standard LTx without EVLP (15% vs 30% *p* = 0.11).^20^ In a recent meta-analysis, the use of EVLP was not associated with increased intensive care unit length of stay, hospital length of stay, ECMO use, or PGD occurrence.^58^ These results are encouraging, but more work is needed to elucidate the ideal donor and recipient characteristics for the application of EVLP.

Recently, there has been new interest in pushing the boundaries of donor lung storage time. Historically, a 6-hour cold ischemic time was proposed as organs with cold ischemic times beyond this point had a lower long-term survival.^59^ But there has been a push to extend the donor lung preservation times to allow for organs to be transported over long distances and avoid overnight transplants, which have been linked to reduced 5-year survival as compared to transplants performed during the day.^60^ Ali et al explored short- and long-term outcomes in lungs that were preserved at 10°C in a small clinical trial and noted no difference in postoperative ECMO, intensive care unit length of stay, hospital length of stay, or 1-year survival.^61^ These findings suggest that extended preservation protocols could expand regional donor pools and improve outcomes by having transplants conducted during more regular work hours.

## Conclusion

Herein, we have reviewed the major strategies to combat the organ shortage, which is the greatest limitation to lung transplantation. Strategies such as the use of extended criteria and DCD donors with the addition of EVLP, and use of HCV- and HIV-positive donor organs are new strategies to expand the donor pool for children and which the authors have begun to implement. Because much of the success has been seen in the adult population, more research is needed to appraise the feasibility and efficacy in the pediatric population. Advancements in surgical techniques and living donor organs are strategies in their infancy, which may also address the need. Furthermore, we should be cognizant of active donor management, PGD, and prevention strategies that significantly impact allograft survival. As pediatric lung transplantation techniques advance, continued scientific inquiry and cooperation across medical specialties will be crucial to maintain safety standards, improve effectiveness, and ensure fair distribution of these vital organs for children in need.

## Author contributions

**Darren Turner**: Conceived the idea for the review, conducted the literature search, and wrote the initial draft of the manuscript. **David L. Morales**: Helped revise sections related to data interpretation and contributed significantly to the final manuscript. **Don Hayes**: The senior advising author of the paper. Provided additional ideas for presentation in the manuscript and contributed significantly to the final manuscript. All authors reviewed and approved the final manuscript and agreed to its submission to the Journal of Heart and Lung Transplantation Open.

## Disclosure statement

The authors declare that they have no known competing financial interests or personal relationships that could have appeared to influence the work reported in this paper.
